# Estimating the number of people who inject drugs in Australia

**DOI:** 10.1186/s12889-017-4785-7

**Published:** 2017-09-29

**Authors:** Sarah Larney, Matthew Hickman, Rebecca Guy, Jason Grebely, Gregory J. Dore, Richard T. Gray, Carolyn A. Day, Jo Kimber, Louisa Degenhardt

**Affiliations:** 1 National Drug and Alcohol Research Centre, UNSW Sydney, Sydney, Australia; 2 Bristol Medical School, University of Bristol, Bristol, UK; 3 Kirby Institute, UNSW Sydney, Sydney, Australia; 4 Sydney Medical School, University of Sydney, Sydney, Australia

**Keywords:** People who inject drugs, Population size, Epidemiology, Multiplier methods, Indirect prevalence estimation

## Abstract

**Background:**

Injecting drug use is associated with considerable morbidity and mortality. Estimates of the size of the population of people who inject drugs are critical to inform service planning and estimate disease burden due to injecting drug use. We aimed to estimate the size of the population of people who inject drugs in Australia.

**Methods:**

We applied a multiplier method which used benchmark data (number of people in opioid substitution therapy (OST) on a snapshot day in 2014) and multiplied it by a factor derived from the prevalence of current OST among people who inject drugs participating in the Australian Needle and Syringe Program Survey in 2014. Estimates of the total population of people who inject drugs were calculated in each state and territory and summed to produce a national estimate. We used the sex and age group distribution seen in datasets relating to people who inject drugs to derive sex- and age-stratified estimates, and calculated prevalence per 1000 population.

**Results:**

Between 68,000 and 118,000 people aged 15–64 years inject drugs in Australia. The population prevalence of injecting drug use was 6.0 (lower and upper uncertainty intervals of 4.3 and 7.6) per 1000 people aged 15–64 years. Injecting drug use was more common among men than women, and most common among those aged 35–44 years. Comparison of expected drug-related deaths based on these estimates to actual deaths suggest that these figures may be underestimates.

**Conclusions:**

These are the first indirect prevalence estimates of injecting drug use in Australia in over a decade. This work has identified that there are limited data available to inform estimates of this population. These estimates can be used as a basis for further work estimating injecting drug use in Australia.

**Electronic supplementary material:**

The online version of this article (10.1186/s12889-017-4785-7) contains supplementary material, which is available to authorized users.

## Background

Injecting drug use is an important risk factor for HIV and viral hepatitis infections [1], and is associated with considerable mortality due to overdose and infectious diseases [2]. Knowledge of the size of the population of people who inject drugs is critically important for understanding burden of infectious disease and other injecting-related harms, as well as planning harm reduction and treatment service provision for this population [3, 4].

Injecting drug use in Australia has previously been estimated to inform projections of hepatitis C virus (HCV) prevalence and burden [5–7]. The most recent of these exercises estimated that in 2005 there were 128,000–294,000 regular (injecting for at least 12 months, multiple times per month, in most months) and occasional (injected in the past 12 months, but not frequently enough to be a regular injector) injectors [7]. This estimate was derived by taking an earlier estimate of the population (from 1997, obtained via a Delphi process [5]), and modelling trends over time consistent with trends in household survey and indicator data, including opioid-related mortality; ambulance attendances and hospitalisations; drug related arrests; HCV notifications; and needle and syringe distribution [7]. By modelling HCV incidence among this population, it was estimated that 206,000–318,000 Australians were HCV antibody positive in 2005 [7].

Updated estimates of injecting drug use in Australia are needed. The federal government has heavily subsidised antiviral therapy for all people with chronic HCV infection, regardless of disease stage or injecting drug use [8, 9]. A clearer understanding of population prevalence of injecting will inform efforts to evaluate progress towards HCV elimination, as well as provide data to assess coverage of drug treatment and harm reduction services. Therefore, we aimed to estimate the number of people who inject drugs in Australia, by sex, age group and state/territory.

## Methods

Indirect prevalence estimation methods are frequently used to estimate ‘hidden’ populations such as people who inject drugs [3]. The most commonly used approach is the multiplier method [3]. This method uses benchmark data that enumerate, at a population level, a behaviour or outcome associated with injecting drug use. The benchmark data are multiplied by a factor derived from the prevalence of that behaviour or outcome in a sample of people who inject drugs, giving an estimate of the total population [10]. This method assumes that the prevalence of a specified behaviour or outcome in people who inject drugs can be estimated with reasonable certainty. However, as a hidden population, it is not possible to obtain a completely random sample of people who inject drugs. As such, multipliers may not reflect the true distribution of the behaviour or outcome of interest, therefore under- or overestimating the true population size. Despite this limitation, multiplier methods are widely used and considered an appropriate method for estimating people who inject drugs [3].

### Population to be estimated

We aimed to estimate the number of people who had injected drugs at least once in the past 12 months, with stratification by sex, age group, and state or territory (hereafter referred to as ‘state’ for simplicity).

### Data sources

We sourced potential benchmark data from multiple data custodians within the Australian Institute of Health and Welfare, the Australian Bureau of Statistics, the NSW Ministry of Health, and the NSW Bureau of Crime Statistics and Research. Benchmark data that were investigated for this estimation exercise are summarised in Table 1. HIV and HCV notifications data were not considered for use as benchmarks as these do not include indicators of recent injecting, and in the case of HIV, prevalence is very low among people who inject drugs [11].

**Table 1:** Data sources investigated to inform estimates of people who inject drugs in Australia

	Benchmark dataset	Indicator	Outcome of multiplier search
New South Wales-specific datasets	Pharmaceutical Drugs of Addiction System	Number of people in opioid substitution therapy at July 1, 2014 (also available from National Opioid Pharmacotherapy Statistics Annual Data Collection)	Multiplier data available from Australian Needle and Syringe Program Survey
	Needle and Syringe Program data	Number of needles and syringes distributed by public needle and syringe programs and pharmacies in NSW, 2014	Data related to number of needle and syringe units distributed rather than individuals accessing needles and syringes. A two-step estimation process that used published data to construct a benchmark number of people accessing needle and syringe programs, followed by standard multiplier method, produced implausibly low estimates
	Re-offending Database	Number of persons proceeded against for use/possess amphetamine, cocaine or narcotics, 2014	Unable to identify suitable multiplier (arrested/charged with use/possess amphetamine, cocaine or opioids in the previous 12 months).
	New South Wales Ambulance	Number of ambulance attendances where naloxone was administered, 2014	Unable to identify suitable multiplier (had an overdose in the past 12 months where an ambulance attended and administered naloxone). The Illicit Drug Reporting System collects data on treatment responses to overdose, but it was not possible to construct a multiplier referring specifically to ambulance-administered naloxone.
	Emergency Department Data Collection	Emergency department presentations for amphetamine, cocaine or opioid overdose, 2014	Unable to identify suitable multiplier (attended an emergency department with amphetamine, cocaine or opioid overdose in the past 12 months)
	Admitted Patients Data Collection	Hospital separations for amphetamine, cocaine or opioid overdose, 2014	Unable to identify suitable multiplier (admitted to hospital with amphetamine, cocaine or opioid overdose in the previous 12 months)
National datasets	National Opioid Pharmacotherapy Statistics Annual Data Collection	Number of people in opioid substitution therapy on a snapshot day in Australia and all states and territories, 2014	Multiplier data extracted from Australian Needle and Syringe Program Survey
	Australian Bureau of Statistics mortality data	Number of amphetamine-, cocaine- and opioid-induced deaths in Australia and all states and territories, 2014	An increasing proportion of opioid-related deaths in Australia are due to pharmaceutical opioids and may be among people who do not inject drugs; hence there were concerns that the benchmark data may not represent the population of people who inject drugs and use of these data would overestimate people who inject drugs. Mortality data were used for validation and limitations of this are noted in the Discussion.

A literature search was undertaken to identify multipliers to apply to the benchmark data (Table 1). With the exception of data relating to opioid substitution therapy (OST), we were unsuccessful in identifying suitable multipliers for the benchmark data, or had concerns about the applicability of the benchmark data to the population of people who inject drugs (Table 1).

Thus, we used benchmark and multiplier data relating to OST to construct these estimates. Benchmark data on the number of people in OST aged 15–64 years on a ‘snapshot day’ in 2014 were available for each state through the National Opioid Pharmacotherapy Statistics Annual Data (NOPSAD) Collection. Data in the NOPSAD Collection are collected by state Departments of Health at the jurisdiction level; data at the sub-state level are not readily available and in some cases not available at all. Methods of data collection vary by jurisdiction due to differences in legislation and resources but generally, all people receiving an OST dose on a given day are counted, either through manual data entry or electronic administrative data collections [12].

State-level multipliers for the OST data were derived from data collected for the 2014 Australian Needle and Syringe Program Survey (ANSPS) [11]. The ANSPS is an annual survey that monitors prevalence of blood borne viral infections and injecting-related risk behaviours among people who inject drugs. During the recruitment period of 1–2 weeks per year, all NSP attendees at participating sites are invited by NSP staff to take part in the ANSPS. All consenting attendees complete a self-administered questionnaire and provide a capillary blood sample for blood borne virus screening. The 2014 ANSPS recruited 2378 respondents from 50 NSP sites in all Australian states; the response rate was 48% [11]. Respondents were asked if they are currently in OST, matching our benchmark data of number of people currently in OST. State-level multipliers (calculated as the inverse of the proportion of ANSPS respondents currently in OST) are shown in Table 2.

**Table 2:** Benchmark and multiplier data used in estimating people who inject drugs in Australia

Area	Number of people aged 15–64 years in OST on a snapshot day, 2014	Number of 2014 ANSPS participants	Proportion (95% CI) of people in 2014 ANSPS reporting current OST	Multiplier (95% CI)
New South Wales	19,246	761	0.42 (0.39, 0.46)	2.38 (2.17, 2.56)
Victoria	14,175	436	0.51 (0.46, 0.56)	1.96 (1.79, 2.17)
Queensland	6362	490	0.30 (0.26, 0.34)	3.33 (2.94, 3.85)
Western Australia	3383	225	0.31 (0.25, 0.37)	3.23 (2.70, 4.00)
South Australia	3165	228	0.37 (0.31, 0.44)	2.70 (2.27, 3.23)
Tasmania	686	69	0.46 (0.34, 0.59)	2.17 (1.69, 2.94)
Australian Capital Territory	917	99	0.53 (0.42, 0.63)	1.89 (1.59, 2.38)
Northern Territory	154	70	0.23 (0.13, 0.34)^a^	5.07 (3.14, 9.47)^a^

*ANSPS* Australian Needle and Syringe Programme Survey, *OST* opioid substitution therapy, *CI* confidence interval

^a^Proportion and multiplier based on five-year moving average due to low numbers in benchmark and multiplier data sources

### Combining benchmark and multiplier data

It is likely that not all people in OST will have injected drugs in a given year. We identified unpublished data from a study of OST patients [13] showing that 57.4% (95% CI 49.7%, 64.8%) of OST patients have injected drugs at least once in the previous six months. We were unable to identify any data on past 12-month injecting drug use while in OST. If we adjust our benchmark data using this percentage, then apply the multiplier, this can be assumed to be a plausible lower bound of the number of people who injected drugs in a year; i.e.

$$ Lower uncertainty interval\ (UI)= benchmark\times 0.574\times multiplier $$

If we assume that *all* people in OST have injected in the past 12 months, we can assume this to be an upper bound of the number of people who inject drugs; i.e.

$$ Upper\  UI= benchmark\times multiplier $$

The mid-point of the lower and upper UI was used as the point estimate of people who inject drugs.

These formulae were used to calculate state-level estimates, which were summed to give a national estimate.

### Stratified estimates

To derive sex- and age-group estimates, we extracted the sex and age group (15–24 years, 25–34 years, 34–44 years, 45–54 years, 55–64 years) distributions of all potential benchmark and multiplier data sources. The extracted proportions were combined in random effects meta-analysis models to derive summary proportions (Additional file 1: Table S1). The national estimate was multiplied by the summary proportion to give sex- and age-group specific estimates.

### Population prevalence of injecting drug use

Denominators for all prevalence estimates were obtained from publicly available Australian Bureau of Statistics data tables for 2014 [14]. Prevalence was calculated per 1000 men/women/persons aged 15–64 years (or specific age group, for age group estimates).

### Validation

To assess the validity of these estimates, we used data on the number of drug-related deaths that may potentially be related to injecting drug use; that is, deaths with an underlying cause of opioid, amphetamine or cocaine poisoning. The number of these deaths among people aged 15–64 years in 2014 was provided by the Australian Bureau of Statistics. Assuming an annual drug-related mortality rate of 0.53% (95% CI: 0.27%, 0.92%) (derived from a cohort study of people who inject drugs in Melbourne, Victoria [15]), we calculated the number of expected opioid, amphetamine or cocaine poisoning deaths based on our estimated population, and compared this to the number of actual drug-related deaths.

## Results

An estimated 93,000 (lower and upper UI of 68,000 and 118,000) people aged 15–64 years inject drugs in Australia, for a population prevalence of 6.0 per 1000 aged 15–64 years (lower and upper UI of 4.3 and 7.6 per 1000 aged 15–64 years) (Table 3). Of these, we estimate that 63,500 are male (lower and upper UI of 46,000 and 80,500) and 30,000 female (lower and upper UI of 22,000 and 38,000). The majority (73%) of people who inject drugs are estimated to be over the age of 35 years, with injecting drug use most prevalent among those aged 35–44 years (11.0 per 1000 aged 35–44 years; lower and upper UI of 8.0 and 13.9 per 1000 aged 35–44 years) (Table 3).

**Table 3:** Estimates of injecting drug use in the past 12 months in Australia, by sex, age group, and state/territory, 2014

	Estimated number of people who inject drugs			Prevalence of injecting drug use per 1000 of population		
	Lower UI	Estimate	Upper UI	Lower UI	Estimate	Upper UI
All persons	68,000	93,000	118,000	4.3	6.0	7.6
Sex						
Male	46,000	63,500	80,500	5.9	8.1	10.3
Female	22,000	30,000	38,000	2.8	3.8	4.9
Age group						
15–24 years	2500	3500	4500	0.9	1.2	1.5
25–34 years	16,500	22,500	28,500	4.7	6.4	8.2
35–44 years	26,000	35,500	45,000	8.0	11.0	13.9
45–54 years	17,000	23,500	29,500	5.5	7.5	9.5
55–64 years	6000	8500	10,500	2.3	3.1	4.0
State/territory						
New South Wales	26,500	36,000	46,000	5.3	7.3	9.3
Victoria	16,000	22,000	27,500	4.1	5.6	7.1
Queensland	12,000	16,500	21,000	3.9	5.3	6.8
Western Australia	6500	8500	11,000	3.6	4.9	6.2
South Australia	5000	6500	8500	4.5	6.1	7.8
Tasmania	<1000	1000	1500	2.6	3.6	4.5
Australian Capital Territory	1000	1500	1500	3.7	5.1	6.4
Northern Territory	<500	500	1000	2.6	3.5	4.5

*UI* Uncertainty interval. Note that sex and age group estimates may not sum to the total estimate due to rounding

Assuming an annual drug-related mortality rate of 0.53% (95% CI: 0.27%, 0.92%) among people who inject drugs, [15] and applying this rate to the national estimate, we would expect between 360 and 626 drug-induced deaths that are potentially related to injecting drug use. There were 846 such deaths in Australians aged 15–64 years in 2014 [16]. Although not all of these deaths would be related to injecting drug use (given recent increases in pharmaceutical opioid use and fatalities among people without a history of injecting drug use [17]), the number of actual opioid-, amphetamine-, or cocaine-induced deaths is greater than the range that would be expected from our estimates. This suggests that either the presented estimates are minimum estimates of injecting drug use Australia, or the drug-related mortality rate used in the validation exercise is not generalizable to the national population of people who inject drugs.

## Discussion

We used multiplier methods to estimate that there are 68,000–118,000 people aged 15–64 years who inject drugs in Australia. This equates to 4–8 people who inject drugs per 1000 people aged 15–64 years. Results of the validation exercise suggest this may be a minimum estimate of people who inject drugs in Australia.

The most recent indirect estimates of people who inject drugs in Australia were for 2005, when it was estimated that there were 128,000–294,000 regular and occasional injectors [7]. This is greater than the estimates presented here, although the 2005 estimate of *regular* injectors (approximately 50,000–110,000) [7] closely overlaps our estimate of the total population injecting in the past 12 months. Crucially, the consensus estimate from which the 2005 estimate was derived was not validated, and therefore its veracity cannot be assessed. Over- or under-estimation associated with the original consensus estimate would lead to over- or under-estimation of people who injected drugs in 2005. This may explain some of the discrepancy between the 2005 estimate and the estimates presented here; further examination of trends over time using the methods described here would also shed light on this issue.

### Limitations

We acknowledge that our approach did not incorporate uncertainty around the number of people in the ANSPS who reported current OST, or uncertainty around the proportion of OST clients who injected drugs. Sex- and age-group estimates did not incorporate the uncertainty around the derived sex and age distributions. We were reluctant to simulate 95% confidence intervals that combined these sources of uncertainty, as this may give spurious precision. As presented, the uncertainty interval around our overall estimate is wide, with the upper bound nearly double the lower bound. Sub-population estimates are similarly presented with wide uncertainty intervals. Future work in this area, as discussed below, will use more sophisticated approaches that account for these various sources of uncertainty. Future work may also incorporate estimation at greater levels of geographical granularity, and over time.

One possible source of bias affecting these estimates is the potential under-representation of people who primarily inject methamphetamine in the data used to generate the multiplier. People who primarily or solely inject methamphetamine typically inject less than daily [18, 19], in comparison to daily or almost daily injecting among people who primarily inject opioids. As such, they are potentially less likely to be recruited to the ANSPS. Limited coverage of rural and regional areas in the ANSPS may also result in under-sampling of methamphetamine users in comparison to their true prevalence in the population of people who inject drugs. These factors would artificially lower the multiplier used to derive the estimates, thereby underestimating people who inject drugs.

There is potentially a population of people who inject drugs who are not in contact with NSP and instead obtain needles and syringes from pharmacies or other sources; if this population is large, then the generalisability of our findings is further limited. However, pharmacies distribute only 11–13% of the total needles and syringes distributed in Australia, with the remainder distributed by NSP [20]. This suggests that most people who inject drugs who obtain needles and syringes via formal avenues of distribution do so through NSP. This does not discount the potential for a population of people who inject drugs who acquire their needles and syringes through other means, such as from peers, but there are no data available to suggest the potential size or demographic profile of such a group.

We reviewed various options to determine an appropriate drug-related mortality rate for use in the validation exercise. No national mortality rates for injecting drug use exist for Australia. A global drug-related mortality rate derived from a systematic review [2] was not considered appropriate due to low HIV prevalence in Australians who inject drugs compared to their counterparts elsewhere [21]. Mortality rates are available for opioid-using cohorts [22], but these may not reflect mortality in the broader injecting population. The selected drug-related mortality rate, from an urban cohort of people who inject drugs, followed between 2008 and 2012 [15], was considered more likely to represent the true mortality rate than any other identified option. However, it is not possible to assess this assumption.

## Conclusions

We have used relatively simple methods to generate these estimates of people who inject drugs. A more sophisticated approach to population estimation, multi-parameter evidence synthesis, has been developed in recent years. This approach aims to generate a single coherent model of all available evidence on both the prevalence of the population and frequency of harm in the population – combining information from administrative data sets and surveys of people who inject drugs to produce a population estimate that is consistent with the evidence [23]. Considerable time and resources are needed to complete such work, but this should be undertaken given the policy and program significance of these estimates.

Using multiplier methods, we have estimated that there are between 68,000 and 118,000 people who inject drugs in Australia. These numbers likely represent a minimum estimate of injecting drug use and can be used as a basis for further work in this area.

## Additional files

Additional file 1: Table S1. Sex and age distributions of people who inject drugs observed in routinely collected and survey data, 2014, and summary proportions obtained by random effects meta-analysis. Sex and age distributions observed in the National Opioid Pharmacotherapy Statistical Annual Data Collection, Australian Bureau of Statistics Causes of Death data, Australian Needle and Syringe Program Survey, and Illicit Drug Reporting System, and summary proportions used to disaggregate the national estimate of people who inject drugs by sex and age. (PDF 73 kb)
