# Supplementary material for: Estimating the number of people who inject drugs in Australia
Source: BMC Public Health. 2017 Sep 29;17:757. doi: 10.1186/s12889-017-4785-7 (PMC5622458; doi:10.1186/s12889-017-4785-7)
Supplement: Supplementary file 1 — Sex and age distributions of people who inject drugs observed in routinely collected and survey data, 2014, and summary proportions obtained by random effects meta-analysis. Sex and age distributions observed in the National Opioid Pharmacotherapy Statistical Annual Data Collection, Australian Bureau of Statistics Causes of Death data, Australian Needle and Syringe Program Survey, and Illicit Drug Reporting System, and summary proportions used to disaggregate the national estimate of people who inject drugs by sex and age. (PDF 73 kb) [file 12889_2017_4785_MOESM1_ESM.pdf]

Supplementary table 1: Sex and age distributions of people who inject drugs observed in routinely collected and survey data, 2014, and summary proportions obtained by random effects meta-analysis

Sub-population	Opioid substitution therapy[1]		Drug-related deaths[2]		Australian Needle and Syringe Program Survey[3]		Illicit Drug Reporting System[4]		
**Age group in years**	**N=48,088**		**N=846**		**N=2,363**		**N=896**		**Summary proportion (95% CI)**
	**n**	**Proportion (95% CI)**	**n**	**Proportion (95% CI)**	**n**	**Proportion (95% CI)**	**n**	**Proportion (95% CI)**	
15-24	1,191	0.02 (0.02, 0.03)	35	0.04 (0.03, 0.06)	142	0.06 (0.05, 0.07)	29	0.03 (0.02, 0.05)	0.04 (0.02, 0.06)
25-34	12,189	0.25 (0.25, 0.26)	173	0.20 (018, 0.23)	591	0.25 (0.23, 0.27)	207	0.23 (0.20, 0.26)	0.24 (0.22, 0.26)
35-44	18,403	0.38 (0.38, 0.39)	295	0.35 (0.32, 0.38)	914	0.39 (0.37, 0.41)	353	0.39 (0.37, 0.39)	0.38 (0.37, 0.39)
45-54	11,717	0.26 (0.24, 0.29)	223	0.26 (0.24, 0.29)	569	0.24 (0.22, 0.26)	241	0.27 (0.24, 0.30)	0.25 (0.24, 0.26)
55-64	4,588	0.10 (0.09, 0.10)	120	0.14 (0.12, 0.17)	147	0.06 (0.05, 0.07)	66	0.07 (0.06, 0.09)	0.09 (0.07, 0.12)
**Sex**	**N=48,269**		**N=846**		**N=2,360**		**N=893**		**Summary proportion (95% CI)**
	**n**	**Proportion (95% CI)**	**n**	**Proportion (95% CI)**	**n**	**Proportion (95% CI)**	**n**	**Proportion (95% CI)**	
Female	16,866	0.35 (0.35, 0.35)	259	0.31 (0.28, 0.34)	725	0.31 (0.29, 0.33)	275	0.31 (0.28, 0.34)	0.32 (0.29, 0.35)
Male	31,403	0.65 (0.65, 0.65)	587	0.69 (0.66, 0.72)	1,635	0.69 (0.67, 0.71)	618	0.69 (0.65, 0.71)	0.68 (0.65, 0.71)

[1]AIHW. National Opioid Pharmacotherapy Statistical Annual Data (NOPSAD) Collection. Canberra: Australian Institute of Health and Welfare, 2015. N varies between age group and sex distribution due to missing data

[2]Australian Bureau of Statistics customised report

[3]Iversen J, Maher L. Australian NSP Survey 20 Year National Data Report 1995-2014. Sydney: Kirby Institute, UNSW Sydney, 2015. N varies between age group and sex distribution due to missing data

[4]Stafford J, Burns L. Australian Drug Trends 2014: Findings from the Illicit Drug Reporting System. Sydney: National Drug and Alcohol Research Centre, UNSW Sydney, 2015. N varies between age group and sex distribution due to missing data
